# Supplementary material for: The impact of college students’ physical exercise on negative emotion: The chain mediating role of self-efficacy and smartphone addiction
Source: PLoS One. 2026 Feb 5;21(2):e0338382. doi: 10.1371/journal.pone.0338382 (PMC12875471; doi:10.1371/journal.pone.0338382)
Supplement: Attachment — Submitted filename: Point-by-point response.docx [file pone.0338382.s002.docx]

**Point-by-point Responses to Reviewer 1**

Dear Editor and dear reviewers

Re: Manuscript Number: PONE-D-25-38262 and Title: The Impact of College Students' Physical Exercise on Negative Emotion: The Chain Mediating Role of Self-efficacy and Smartphone Addiction

Thank you very much for your comments and professional advice. These opinions help to improve the academic rigor of our manuscript. Based on your suggestion and request, we have made corrected modifications to the revised manuscript. Here are point-by-point responses to your comments. We hope that our work can be improved again. Furthermore, we would like to show the details as follows:

Sincerely,

On behalf of all authors

**Comment #1:**

Expand the Introduction:

-Provide a more detailed overview of the existing literature on the relationships between physical exercise, negative emotions, self-efficacy, and smartphone addiction.

-Clearly articulate the gaps in the literature that the current study aims to address.

-Consider adding a section on the specific cultural context of college students in China and how it may influence the relationships between the variables.

-Elaborate on the rationale for choosing Henan and Jiangsu provinces.

**Response#1:**

Thank you for your valuable suggestions regarding the introduction. We have comprehensively revised the introduction in accordance with your recommendations. In response to the suggestion to ‘provide a more detailed overview of the existing literature,’ we have expanded the literature review on the relationship between physical exercise, negative emotions, self-efficacy, and smartphone addiction. Specific modifications can be found in lines 91-99 and 155-161 and 193-200 of the manuscript. To clarify the research objectives, we have explicitly stated the literature gap addressed by the present study, as detailed in lines 99-109 of the manuscript. Furthermore, we have incorporated a discussion on the cultural context of Chinese university students and its potential influence on the relationships between variables, as outlined in lines 224-232 of the manuscript. We extend our gratitude once more for your guidance. Additionally, we have elaborated on the rationale for selecting Henan and Jiangsu provinces, as explained in lines 233-248. We extend our gratitude once more for your guidance.

**Comment #2:**

Strengthen the Theoretical Framework:

-Provide a more in-depth discussion of Bandura's Social Cognitive Theory (SCT) and how it applies to the relationships between the variables.

-Explain how SCT can help explain the mediating roles of self-efficacy and smartphone addiction.

-Consider discussing other relevant theories, such as the self-determination theory, to provide a more comprehensive theoretical framework.

**Response#2:**

Thank you for your valuable suggestions regarding the theoretical framework. We have substantially refined the theoretical framework in the introduction as suggested. Addressing the recommendation to ‘discuss Bandura's Social Cognitive Theory (SCT) in greater depth and how it applies to the relationships between variables,’ we have elaborated on SCT's core concepts within the introduction and provided a detailed analysis of how SCT explains the relationship between physical exercise, negative emotions, self-efficacy, and smartphone addiction. Additionally, we have specifically clarified how SCT aids in explaining the mediating role of self-efficacy in smartphone addiction. Furthermore, to provide a more comprehensive theoretical framework, we have incorporated a discussion of Self-Determination Theory (SDT), exploring its complementarity with SCT and how both theories collectively account for the relationships among these variables. Please refer to lines 249-296 of the manuscript. We believe these modifications will further enhance the theoretical depth of the manuscript.

**Comment #3:**

Address the Limitations of the Study:

-Acknowledge the limitations of the cross-sectional design and the inability to draw causal inferences.

-Discuss the potential for recall bias and social desirability bias due to the use of self-report measures.

-Address the limitations of generalizability due to the focus on college students in China.

**Response#3:**

We are grateful for your detailed suggestions regarding the limitations of this study. We have incorporated your recommendations by providing a clear and comprehensive discussion of the study's limitations within the manuscript. We fully acknowledge the limitations inherent in a cross-sectional design and emphasise that this study cannot draw causal inferences. Furthermore, we discuss the potential for recall bias and social desirability bias arising from the use of self-report measures, analysing their possible impact on the findings in the Discussion section. Finally, we explicitly acknowledge the generalisability limitations arising from our focus on Chinese university students within the discussion section. We also propose strategies for future research to enhance the generalisability of findings. Please refer to lines 785-816 of the manuscript. We believe these supplementary clarifications will enhance transparency regarding the study's limitations and provide readers with a more accurate understanding.

**Comment #4:**

Refine the Discussion Section:

-Ensure that all interpretations in the discussion section are clearly linked back to the empirical findings.

-Provide a more nuanced discussion of the implications of the findings for mental health interventions.

-Consider discussing the potential for future research to address the limitations of the current study.

-Make stronger recommendations for interventions based on the results.

**Response#4:**

We are grateful for your valuable suggestions to enhance the discussion section of this study. We have carefully revised the discussion to strengthen its rigour and practical relevance. We have ensured all interpretations within the discussion are closely tied to empirical findings, with interpretations of the results grounded in objective data and statistical analysis. Specific revisions can be found in lines 556-559 and 604-6066 and 660-672 and 682-691 and 720-723 of the manuscript. Additionally, we have provided a more detailed discussion on the implications of our findings for mental health interventions, elaborating on how the results can be utilised to design and implement mental health interventions for university students, as detailed in lines 742-784 of the manuscript. Furthermore, we have explored the potential for future research to address current limitations and proposed specific avenues for further investigation to deepen understanding of related issues, as detailed in lines723-816 of the manuscript. Finally, we have formulated stronger intervention recommendations based on our findings, emphasising the potential benefits of these measures, as outlined in lines 742-784 of the manuscript. We believe these revisions will significantly enhance the depth and practical value of the discussion section.

**Comment #5:**

Formatting and Clarity:

-Ensure that the manuscript is free of grammatical errors and typos.

-Use clear and concise language throughout the manuscript.

-Follow the formatting guidelines of the target journal.

-Improving the flow and coherence of paragraphs to maintain reader engagement.

**Response#5:**

We are grateful for your detailed suggestions regarding the format and clarity of this study. We have undertaken a thorough and meticulous revision of the manuscript in accordance with your recommendations. The entire text has been carefully proofread to ensure the absence of grammatical errors and spelling mistakes, with particular attention paid to achieving clarity and conciseness in linguistic expression. Concurrently, we have strictly adhered to the target journal's formatting guidelines, adjusting the manuscript's layout to meet its requirements. Furthermore, we have enhanced paragraph flow and coherence to clarify the article's logical structure and improve readability, thereby better retaining reader engagement. For specific modifications, please refer to the full manuscript. We believe these improvements will render the paper more accessible and easier to comprehend.

**Comment #6:**

Figures and Tables:

-Ensure that all figures and tables are clearly labelled and easy to understand.

-Provide a brief description of the key findings in the figure and table captions.

-In Table 2, clarify the labels "Risk-free," "Medium risk," and "High risk."

**Response#6:**

Thank you for your specific suggestions regarding the figures and tables in this study. We have carefully reviewed and revised the figures and tables in the manuscript to ensure they are clear and easily understood. We have ensured that all relevant figures and tables are accompanied by clear labels, with their captions providing concise summaries of key findings to enable readers to grasp information swiftly. Please refer to all figures and tables within the manuscript for the specific modifications made. Regarding your suggestion concerning Table 2, the labels ‘Risk-free’, ‘medium risk’ and ‘high risk’ have been clarified; please see lines 898 -902 and 418-422 of the manuscript for details. We believe these amendments will enhance the readability of the figures and tables and improve the accuracy of the information conveyed.

**Comment #7:**

Consider These Points:

-Clarify how the data was collected, ensuring the process is replicable.

-Discuss how multicollinearity was addressed, especially given the high correlations among some variables.

-Explore whether the relationships found differ significantly across demographic groups (e.g., gender, region).

-Discuss the broader implications for universities and policymakers.

-Offer practical guidance for designing effective interventions based on study findings.

**Response#7:**

We are grateful for your additional suggestions regarding this study. We have carefully considered these comments and made further refinements to the manuscript. The specific procedures for data collection have been detailed in the Methods section to ensure the reproducibility of the process; please refer to lines 300-316 of the manuscript for the specific modifications. Regarding the issue of multicollinearity, we have provided corresponding explanations, detailed in lines 449-454 of the manuscript. Furthermore, to explore variations in the identified relationships across different demographic groups (e.g., gender, region), we conducted additional analyses, detailed in lines 485-503 of the manuscript. Additionally, we have discussed the broader implications of our findings for universities and policymakers in the Discussion section, offering corresponding recommendations, detailed in lines 573-599 of the manuscript. Finally, based on our findings, we provide practical guidance for designing effective interventions, as detailed in lines 742-764 of the manuscript. We believe these revisions will enhance the manuscript's completeness and practical utility.

**Point-by-point Responses to Reviewer 2**

Dear Editor and dear reviewers

Re: Manuscript Number: PONE-D-25-38262 and Title: The Impact of College Students' Physical Exercise on Negative Emotion: The Chain Mediating Role of Self-efficacy and Smartphone Addiction

Thank you very much for your comments and professional advice. These opinions help to improve the academic rigor of our manuscript. Based on your suggestion and request, we have made corrected modifications to the revised manuscript. Here are point-by-point responses to your comments. We hope that our work can be improved again. Furthermore, we would like to show the details as follows:

Sincerely,

On behalf of all authors

**Comment #1:**

The introduction section lacks a thorough review of previous research. The authors should conduct a more comprehensive analysis of existing studies, including their strengths and limitations, while clearly articulating the innovative aspects of this study and how it differs from prior research in the field.

**Response#1:**

We appreciate your insightful comment regarding the introduction’s literature review. In response, we have significantly expanded the introduction to provide a more comprehensive analysis of previous research. This includes a more thorough examination of existing studies, highlighting both their strengths and limitations. We have also explicitly articulated the innovative aspects of our study, clarifying how it differs from prior research to the field. Please refer to lines 99-109 and 127-136 and 205-214 of the manuscript.

**Comment #2:**

Given that this study employs a cross-sectional design, special attention should be paid to the wording of hypotheses to avoid implying causal relationships. The language should be carefully framed to describe associations rather than causation throughout the manuscript.

**Response#2:**

Thank you for your valuable feedback on our research. We have carefully considered your comments regarding the study design and wording. In response to your concern about the cross-sectional design, we have meticulously reviewed and revised the wording of our hypotheses and the entire manuscript to ensure that our language accurately reflects the correlational nature of the study, avoiding any implication of causal relationships. We have focused on describing associations rather than causation throughout. Specific revisions can be found throughout the manuscript.

**Comment #3:**

The study's exclusive focus on Eastern and Central China may limit the generalizability of its findings. This geographical limitation should be explicitly acknowledged and discussed in the study's limitations section.

**Response#3:**

Thank you for your insightful comment. We acknowledge the reviewer’s concern regarding the geographical limitations of our study, specifically its focus on Eastern and Central China, and its potential impact on the generalizability of our findings. In response, we have explicitly acknowledged this limitation and discussed its implications within the limitations section of the manuscript. Please refer to the limitations section (lines 803-816) for details.

**Comment #4:**

The discussion would benefit from comparative analysis with similar studies conducted in Western populations, as smartphone addiction patterns may vary due to cultural differences and other factors. We recommend adding a dedicated discussion on potential cultural influences, such as the possibility that Chinese students face greater academic pressure leading to different coping mechanisms.

**Response#4:**

Thank you for your insightful feedback. We appreciate your suggestion to enhance the discussion section with a comparative analysis against studies conducted in Western populations. We recognize that smartphone addiction patterns and associated factors may vary due to cultural differences and other influences. Therefore, we have added a dedicated section to the discussion that specifically addresses potential cultural influences, including the possibility that Chinese students may experience greater academic pressure, potentially leading to different coping mechanisms and affecting the relationships between the variables studied. Please refer to lines 623-631 and 644-653 and 695-702 and 770-777 of the manuscript.

**Comment #5:**

While the conclusion appropriately notes the mental health benefits of physical exercise, it fails to provide practical implementation guidance. The authors should include specific recommendations for intervention strategies at the institutional level, such as how universities might practically apply these findings.

**Response#5:**

Thank you for your insightful comment on the conclusion. We acknowledge the reviewer’s suggestion to enhance the practical implications of our findings. In response, we have revised the conclusion to include specific recommendations for intervention strategies at the institutional level. These recommendations outline how universities could practically apply our findings to promote physical exercise and support students’ mental health. Please refer to lines 742-784 of the manuscript.

**Comment #6:**

The reference formatting requires standardization, as some entries include DOI identifiers while others do not. We recommend ensuring all references follow a consistent format in accordance with the journal's style guidelines.

**Response#6:**

Thank you for pointing out the inconsistencies in the reference format. We have reviewed all references in accordance with the journal's formatting guidelines; however, some references were indeed lacking DOI numbers. Please refer to the revised reference list for details.

**Point-by-point Responses to Reviewer 3**

Dear Editor and dear reviewers

Re: Manuscript Number: PONE-D-25-38262 and Title: The Impact of College Students' Physical Exercise on Negative Emotion: The Chain Mediating Role of Self-efficacy and Smartphone Addiction

Thank you very much for your comments and professional advice. These opinions help to improve the academic rigor of our manuscript. Based on your suggestion and request, we have made corrected modifications to the revised manuscript. Here are point-by-point responses to your comments. We hope that our work can be improved again. Furthermore, we would like to show the details as follows:

Sincerely,

On behalf of all authors

**Comment #1:**

Introduction Enhancement:

While the introduction provides context, expand the literature review to engage with prior studies—particularly those examining specific relationships between physical exercise, self-efficacy, and smartphone addiction—and integrate critical perspectives.

**Response#1:**

Thank you for your comment on the introduction. We have expanded the literature review to more thoroughly engage with prior studies, focusing on those that specifically investigate the relationships between physical exercise, self-efficacy, and smartphone addiction. We have also integrated critical perspectives to offer a more nuanced understanding of the existing research landscape. Please refer to lines 91-109 and 155-161 and 193-200 of the manuscript for the details.

**Comment #2:**

Theoretical Justification:

Hypotheses require stronger grounding in theoretical models and existing literature. Justify the selection of self-efficacy and smartphone addiction as mediators with explicit theoretical rationale.

**Response#2:**

We appreciate your feedback on the theoretical justification of our hypotheses. In the revised manuscript, we have strengthened the grounding of our hypotheses in relevant theoretical models and existing literature. We have provided explicit theoretical rationale for the selection of self-efficacy and smartphone addiction as mediators, explaining how these constructs align with our overall framework. The detailed justifications can be found on lines 167-172 and 214-221 and 266-282.

**Comment #3:**

Psychometric Validation:

Reliability and validity of scales (e.g., MPATS, DASS-21) are insufficiently addressed. Report internal consistency and validity metrics specific to this study’s sample.

**Response#3:**

Thank you for pointing out the need for more detailed psychometric validation. We have addressed this in the revised manuscript by reporting the internal consistency and validity metrics specific to our study’s sample for all scales used, including the MPATS and DASS-21. This information can be found of the manuscript on lines 347-353 and 373-378 and 388-394 and 411-416 and 466-477.

**Comment #4:**

Mediation Analysis Transparency:

Clarify procedural steps for mediation testing, including model selection criteria and bootstrap implementation methodology.

**Response#4:**

We appreciate your suggestion for greater transparency in our mediation analysis. In the revised manuscript, we have clarified the procedural steps for mediation testing, providing detailed information on our model selection criteria and the methodology used for implementing the bootstrap analysis. This information can be found on lines 426-430 of the manuscript.

**Comment #5:**

Discussion Depth:

Strengthen interpretation of chain mediation findings and articulate practical implications for university mental health interventions.

**Response#5:**

Thank you for your comment on the discussion section. We have significantly strengthened the interpretation of the chain mediation findings and expanded on the practical implications for university mental health interventions. Please refer to lines 573-581 and 727-733 of the manuscript for the detailed discussion.

**Comment #6:**

Design Limitations:

Explicitly acknowledge constraints of the cross-sectional design.

**Response#6:**

We acknowledge your point regarding the limitations of our study’s design. In the revised manuscript, we have explicitly acknowledged the constraints of the cross-sectional design. Please refer to lines 787-791 of the manuscript for the detailed discussion.

**Point-by-point Responses to Reviewer 4**

Dear Editor and dear reviewers

Re: Manuscript Number: PONE-D-25-38262 and Title: The Impact of College Students' Physical Exercise on Negative Emotion: The Chain Mediating Role of Self-efficacy and Smartphone Addiction

Thank you very much for your comments and professional advice. These opinions help to improve the academic rigor of our manuscript. Based on your suggestion and request, we have made corrected modifications to the revised manuscript. Here are point-by-point responses to your comments. We hope that our work can be improved again. Furthermore, we would like to show the details as follows:

Sincerely,

On behalf of all authors

**Comment #1:**

Methods section: While the Physical Activity Rating Scale and other measurement tools demonstrated good internal consistency, the study does not provide detailed information regarding the validation of their psychometric properties, within the current sample.

**Response#1:**

We appreciate your feedback regarding the validation of psychometric properties. While our study demonstrated good internal consistency for the Physical Activity Rating Scale and other measurement tools, we acknowledge the need for further clarification. We have revised the methods section to include more details about the validation process of the scales used. Please refer to lines 347-353 and 373-378 and 388-393 and 411-416 of the manuscript for the specific revisions and added details.

**Comment #2:**

Methods section: The study’s sample, though large, shows an uneven distribution of participants across gender and grade levels. This imbalance may affect the generalizability of the findings. The authors are advised to discuss the potential impact of this sampling bias and clarify whether stratified analysis or weighting methods were employed to address these discrepancies.

**Response#2:**

Thank you for pointing out the potential sampling bias in our study. We acknowledge the uneven distribution of participants across gender and grade levels. We clarified whether stratified analysis or weighting methods were employed to address these discrepancies. Please refer to lines 323-329 of the manuscript for the specific revisions and added details.

**Comment #3:**

Methods section: The study relies on self-reported measures for key variables, which may introduce recall or social desirability biases. The authors should elaborate on steps taken to mitigate these biases and discuss the limitations of self-reported data in the context of their findings.

**Response#3:**

Thank you for your insightful comments. We have addressed the concern regarding self-reported measures and the potential for recall and social desirability biases. We have elaborated on the strategies implemented to minimize these biases. Please refer to lines 454-466 of the manuscript for the specific revisions and added details. Furthermore, we have added a dedicated discussion of the limitations inherent in self-reported data, acknowledging how these limitations might influence the interpretation of our findings. For more detailed information, please refer to lines 791-803 of the manuscript.

**Comment #4:**

Discussion section: The study employs a cross-sectional design, which inherently limits the ability to establish causal relationships between variables. While the chain mediation model is compelling, the authors should explicitly acknowledge this limitation and recommend longitudinal or experimental designs for future research to validate causality.

**Response#4:**

Thank you for highlighting the limitations of the cross-sectional design. We fully acknowledge that the cross-sectional nature of our study restricts our ability to definitively infer causal relationships between the variables. We have revised the discussion section to explicitly state this limitation and to temper our interpretations accordingly. Furthermore, we have included specific recommendations for future research, advocating for the use of longitudinal or experimental designs to explore the causal pathways suggested by our chain mediation model. For further details on these revisions, please refer to line 787-791 of the manuscript.

**Comment #5:**

Discussion section: While the results are thoroughly presented, the discussion could benefit from a deeper theoretical integration.

**Response#5:**

We appreciate your feedback regarding the discussion’s theoretical integration. We have further clarified the theoretical underpinnings of our observations and providing a more comprehensive interpretation. You can find these revisions and expansions in lines 623-631 and 655-669 and 678-708 and 777-784 of the manuscript.

**Comment #6:**

Discussion section: The authors note that physical exercise may sometimes correlate positively with negative emotions due to emotional venting.

**Response#6:**

Thank you for drawing our attention to this important nuance regarding the potential positive correlation between physical exercise and negative emotions, often attributed to emotional venting. We have expanded upon this point in the discussion section, providing a more detailed explanation of this phenomenon. For a more thorough discussion of this aspect, please refer to lines 632-653 of the manuscript.

**Comment #7:**

Limitations: The study mentions potential biases from questionnaire responses but does not address the lack of objective measures. Including these limitations would provide a more balanced perspective on the study’s constraints.

**Response#7:**

Thank you for pointing out the need for a more balanced perspective regarding the study’s limitations. We have revised the limitations section to explicitly address the absence of objective measures in our study. For a detailed account of these additions, please refer to lines 791-803 of the manuscript.
