# Supplementary material for: The impact of college students’ physical exercise on negative emotion: The chain mediating role of self-efficacy and smartphone addiction
Source: PLoS One. 2026 Feb 5;21(2):e0338382. doi: 10.1371/journal.pone.0338382 (PMC12875471; doi:10.1371/journal.pone.0338382)
Supplement: Attachment — Submitted filename: Point-by-point_response_auresp_2.docx [file pone.0338382.s003.docx]

**Point-by-point Responses to Reviewer 2**

Dear Editor and dear reviewers

Re: Manuscript Number: PONE-D-25-38262 and Title: The Impact of College Students' Physical Exercise on Negative Emotion: The Chain Mediating Role of Self-efficacy and Smartphone Addiction

Thank you very much for your comments and professional advice. These opinions help to improve the academic rigor of our manuscript. Based on your suggestion and request, we have made corrected modifications to the revised manuscript. Here are point-by-point responses to your comments. We hope that our work can be improved again. Furthermore, we would like to show the details as follows:

Sincerely,

On behalf of all authors

**Comment #1:**

one reviewer still has doubts about the sampling method of this study; it does not affect the main conclusions of the research. Authors should resolve that.

**Response#1:**

We sincerely appreciate this valuable feedback from the reviewers. This study employed the “multistage stratified cluster sampling” method widely used in large-scale social surveys. The design aimed to maximize the representativeness of the sample for China's college student population within limited resources. The sampling process comprised three distinct stages: First, we purposefully selected Jiangsu Province in East China and Henan Province in Central China based on their representativeness in economic development and educational resources, seeking to encompass students from diverse socioeconomic backgrounds; Second, within each province, we randomly selected a total of 8 institutions encompassing both undergraduate and vocational college levels. Third, at each selected institution, we stratified by academic year and major, then randomly clustered classes as survey units. This design systematically accounts for multiple dimensions—region, institution type, academic year, and major—to construct a logically structured sample framework.

We fully acknowledge that despite this design, the final sample exhibits some imbalance in gender distribution (60.5% female) and grade level (46.3% sophomore), which is indeed difficult to completely avoid in large-scale field surveys. Therefore, during the data analysis phase, we proactively implemented two statistical measures to correct potential biases arising from these factors: First, we applied weighting to the data based on gender and grade distribution to enhance the sample's representativeness of the population. Second, in all subsequent key regression analyses and mediation effect tests, we included demographic variables such as gender, grade, region, and age as control variables in the models. This ensures that the core relationships we report—between physical exercise, self-efficacy, smartphone addiction, and negative emotions—represent net effects after controlling for these demographic background factors.

We extend our gratitude once more to the reviewers for their meticulous scrutiny, which prompted deeper reflection and clarification of our methodology. We believe the above explanations effectively address concerns regarding sampling methods and further strengthen the theoretical foundation of our study's conclusions.
